# Genetic Characterization of Type A Enterotoxigenic *Clostridium perfringens* Strains

**DOI:** 10.1371/journal.pone.0005598

**Published:** 2009-05-19

**Authors:** Agi Deguchi, Kazuaki Miyamoto, Tomomi Kuwahara, Yasuhiro Miki, Ikuko Kaneko, Jihong Li, Bruce A. McClane, Shigeru Akimoto

**Affiliations:** 1 Department of Microbiology, Wakayama Medical University School of Medicine, Wakayama, Japan; 2 Department of Bacteriology, The University of Tokushima Faculty of Medicine, Tokushima, Japan; 3 Department of Microbiology and Molecular Genetics, University of Pittsburgh School of Medicine, Pittsburgh, Pennsylvania, United States of America; Charité - Universitätsmedizin Berlin, Germany

## Abstract

*Clostridium perfringens* type A, is both a ubiquitous environmental bacterium and a major cause of human gastrointestinal disease, which usually involves strains producing *C. perfringens* enterotoxin (CPE). The gene (*cpe*) encoding this toxin can be carried on the chromosome or a large plasmid. Interestingly, strains carrying *cpe* on the chromosome and strains carrying *cpe* on a plasmid often exhibit different biological characteristics, such as resistance properties against heat. In this study, we investigated the genetic properties of *C. perfringens* by PCR-surveying 21 housekeeping genes and genes on representative plasmids and then confirmed those results by Southern blot assay (SB) of five genes. Furthermore, sequencing analysis of eight housekeeping genes and multilocus sequence typing (MLST) analysis were also performed. Fifty-eight *C. perfringens* strains were examined, including isolates from: food poisoning cases, human gastrointestinal disease cases, foods in Japan or the USA, or feces of healthy humans. In the PCR survey, eight of eleven housekeeping genes amplified positive reactions in all strains tested. However, by PCR survey and SB assay, one representative virulence gene, *pfoA*, was not detected in any strains carrying *cpe* on the chromosome. Genes involved in conjugative transfer of the *cpe* plasmid were also absent from almost all chromosomal *cpe* strains. MLST showed that, regardless of their geographic origin, date of isolation, or isolation source, chromosomal *cpe* isolates, i) assemble into one definitive cluster ii) lack *pfoA* and iii) lack a plasmid related to the *cpe* plasmid. Similarly, independent of their origin, strains carrying a *cpe* plasmid also appear to be related, but are more variable than chromosomal *cpe* strains, possibly because of the instability of *cpe*-borne plasmid(s) and/or the conjugative transfer of *cpe*-plasmid(s) into unrelated *C. perfringens* strains.

## Introduction

*Clostridium perfringens,* an anaerobic Gram-positive bacterium, is ubiquitous in the intestinal flora of human and animals, and is also commonly isolated from environmental materials such as soil and water [1], [2]. Moreover, *C. perfringens* is an extremely important pathogen of human and domestic animals. In a commonly used classification scheme, *C. perfringens* is divided into five toxinotypes (A to E) based on the production of four toxins (alpha, beta, epsilon, and iota); however, this bacterium also produces ten other toxins such as *C. perfringens* enterotoxin (CPE), beta2 toxin, and theta toxin (also known at perfringolysin O or PFO) [2].

Of these many toxins, CPE is extremely important for human gastrointestinal diseases such as food poisoning and antibiotic-associated diarrhea [2]. Interestingly, the gene encoding this toxin (*cpe*) is highly conserved, although the *cpe* gene can be present on the chromosome or on a large plasmid(s) in type A strains [3]. Plasmid *cpe*-carrying strains can cause *C. perfringens* food poisoning outbreaks, but chromosomal *cpe* isolates are responsible for the majority of foodborne illnesses [3]–[5]. In contrast, nearly all CPE-associated human AAD is caused by plasmid *cpe* isolates. In addition, there are several reports of biological differences, such as heat resistance and other traits [6]–[8], between chromosomal *cpe* isolates and plasmid *cpe* isolates that may suggest these two types of strains possess different genetic backgrounds.

To investigate the genetic background of a bacterium, total genome sequence analysis has been performed in many pathogens. To date, the complete genome sequence of three pathogenic type A strains of *C. perfringens* (ATCC13124, SM101, and strain 13) has been published [9], [10] and genome sequencing of several animal toxigenic strains (type B, C, and E) is in progress. Thus far, all sequenced *C. perfringens* isolates share a similar chromosomal genetic organization, although this genome sequencing has tested only a limited number of strains. Multilocus sequence typing (MLST) represents an alternative approach capable of investigating the genetic characterization of many strains of a given species. A MLST approach has been applied to many bacteria, but not yet to compare the genetic relatedness of large numbers of chromosomal *cpe* isolates vs. plasmid *cpe* isolates from varied sources [11]–[13].

This study sought to characterize the genetic background of enterotoxigenic *C. perfringens* strains, firstly using a conventional PCR survey for representative virulence genes on the chromosome or for genes borne on toxin plasmid(s), as well as for several housekeeping genes. After confirming selected PCR results by Southern blot, a MLST analysis based on those PCR/Southern blot results was then applied to characterize the genetic backgrounds of chromosomal *cpe* isolates vs. plasmid *cpe* isolates.

## Results

### PCR survey for representative *C. perfringens* chromosomal or plasmid-borne genes

To assess genetic diversity amongst enterotoxigenic *C. perfringens* isolates, a PCR survey was first performed to evaluate the carriage of selected genes including chromosomal toxin genes (*plc* and *pfoA*), several chromosomal housekeeping genes, plasmid maintenance genes, and genes related to plasmid transfer (Table 1).

**Table 1:** The results of PCR survey of the representative genes.

strain	genotype		genes																					
			*cpe*	*cpb2*	*gyrB*	*sigK*	*sodA*	*groEL*	*pgk*	*nadA*	*plc*	*colA*	*lonB*	*eno*	*virS*	*pfoA*	*tcpH*	*tcpF*	*rep*	*cna*	*soj*	*parB*	*topA*	*bcn*
Strain 13	A		−	+	+	+	+	+	+	+	+	+	+	+	+	+	−	−	−	+	+	+	+	−
ATCC13124	A	type strain	−	−	+	+	+	+	+	+	+	+	+	+	+	+	−	−	−	−	−	−	−	−
ATCC3624	A	Gas gangrene	−	−	+	+	+	+	+	+	+	+	+	+	+	+	−	−	−	−	−	−	−	−
KZ210	A	BP6K derivative	−	−	+	+	+	+	+	+	+	+	+	+	+	+	−	−	−	−	−	−	−	+
NCTC8239	A	food poisoning	+	−	+	+	+	+	+	+	+	+	+	−	+	−	−	−	−	−	+	+	+	−
NCTC8798	A	food poisoning	+	−	+	+	+	+	+	+	+	+	+	−	−	−	−	−	−	−	−	−	−	+
OSAKA1	A	food poisoning	+	−	+	+	+	+	+	+	+	+	+	−	−	−	−	−	−	−	+	+	+	−
OSAKA2	A	food poisoning	+	−	+	+	+	+	+	+	+	+	+	−	−	−	−	−	−	−	+	+	+	−
OSAKA3	A	food poisoning	+	−	+	+	+	+	+	+	+	+	+	−	−	−	−	−	−	−	+	+	+	−
OSAKA4	A	food poisoning	+	−	+	+	+	+	+	+	+	+	+	−	−	−	−	−	−	−	+	+	+	−
W4232	A	food poisoning	+	−	+	+	+	+	+	+	+	+	−	−	+	−	−	−	−	−	+	+	+	−
W5837	A	food poisoning	+	−	+	+	+	+	+	+	+	+	+	−	−	−	−	−	−	−	+	+	+	+
W6205	A	food poisoning	+	−	+	+	+	+	+	+	+	+	−	−	−	−	−	−	−	−	+	+	+	−
P-1/09/03	A	food	+	−	+	+	+	+	+	+	+	+	+	+	+	−	−	+	+	+	+	+	+	−
T-1/08/03	A	food	+	−	+	+	+	+	+	+	+	+	+	+	+	−	−	−	−	+	+	+	+	−
F4013	A	sporadic diarrhea	+	+	+	+	+	+	+	+	+	+	+	+	+	+	+	+	+	+	+	+	+	+
F4969	A	sporadic diarrhea	+	−	+	+	+	+	+	+	+	+	+	+	+	+	+	+	+	+	−	−	−	−
F5603	A	sporadic diarrhea	+	+	+	+	+	+	+	+	+	+	+	+	+	+	+	+	+	+	+	+	−	+
No.2	A	food poisoning	+	+	+	+	+	+	+	+	+	+	+	+	+	+	+	+	+	+	+	+	+	−
No.24	A	food poisoning	+	+	+	+	+	+	+	+	+	+	+	+	+	+	+	+	+	+	+	+	+	−
No.110	A	food poisoning	+	+	+	+	+	+	+	+	+	+	+	+	+	+	+	+	+	+	+	+	+	−
T1	A	food poisoning	+	+	+	+	+	+	+	+	+	+	+	+	+	−	+	+	+	+	+	+	+	−
T16	A	food poisoning	+	+	+	+	+	+	+	+	+	+	+	+	+	−	+	+	+	+	+	+	+	−
T102	A	food poisoning	+	+	+	+	+	+	+	+	+	+	+	+	+	−	+	+	+	+	+	+	+	−
BL-D1	A	sepsis	−	+	+	+	+	+	+	+	+	+	+	+	+	+	+	+	+	+	+	+	+	+
DR-T1	A	diarrhea	−	−	+	+	+	+	+	+	+	+	+	+	+	+	−	−	−	−	+	+	+	−
BI-D2	A	cholecystitis	−	−	+	+	+	+	+	+	+	+	+	+	+	+	−	−	−	−	+	+	+	−
MR1-1	A	healthy	−	+	+	+	+	+	+	+	+	+	+	+	+	+	−	−	−	−	+	+	+	−
MR1-2	A	healthy	−	+	+	+	+	+	+	+	+	+	−	+	+	+	+	+	+	+	−	−	−	−
MR2-2	A	healthy	−	+	+	+	+	+	+	+	+	+	+	+	+	+	+	+	+	+	−	−	−	−
MR2-3	A	healthy	−	−	+	+	+	+	+	+	+	+	+	+	+	+	−	−	−	−	−	−	−	−
MR2-4	A	healthy	+	+	+	+	+	+	+	+	+	+	+	+	+	−	+	+	+	+	+	+	+	−
MR2-5	A	healthy	−	−	+	+	+	+	+	+	+	+	+	+	+	+	−	−	−	−	+	+	+	+
MR2-9	A	healthy	−	+	+	+	+	+	+	+	+	+	+	+	+	+	+	+	+	+	+	+	+	+
MR2-12	A	healthy	−	+	+	+	+	+	+	+	+	+	+	+	+	−	+	+	+	+	−	−	−	−
MR2-14	A	healthy	−	−	+	+	+	+	+	+	+	+	+	+	+	+	−	−	−	−	−	−	−	−
MR2-19	A	healthy	−	+	+	+	+	+	+	+	+	+	+	+	+	+	+	+	+	+	+	−	+	−
MR2-20	A	healthy	−	+	+	+	+	+	+	+	+	+	−	+	−	+	+	+	+	+	−	−	−	−
JCM1290	A	ATCC13124	−	−	+	+	+	+	+	+	+	+	+	+	+	+	−	−	−	−	−	−	−	−
JCM3819	A	ATCC3629	−	−	+	+	+	+	+	+	+	+	+	+	+	+	−	−	−	−	−	−	−	−
JCM3816	A	ATCC3624	−	−	+	+	+	+	+	+	+	+	+	+	+	+	−	−	−	−	−	−	−	−
NCTC8533	B	animal disease (lamb)	−	+	+	+	+	+	+	+	+	+	+	+	+	+	+	+	+	+	+	+	+	−
NCTC8081	C	necrotizing enteritis	+	−	+	+	+	+	+	+	+	+	+	−	−	−	−	+	+	+	+	+	+	−
NCTC3182	C	animal disease (sheep)	−	−	+	+	+	+	+	+	+	+	+	+	+	+	+	+	+	+	−	−	−	−
NCTC8346	D	animal disease (sheep)	−	−	+	+	+	+	+	+	+	+	+	+	−	+	+	+	+	+	−	−	−	+
NCTC8084	E	animal disease (calf)	−	+	+	+	+	+	+	+	+	+	+	+	+	+	+	+	+	+	+	−	+	+
M-01	A	food	−	−	+	+	+	+	+	+	+	+	+	−	+	−	−	−	+	+	−	−	−	+
M-02	A	food	−	−	+	+	+	+	+	+	+	+	+	+	+	+	+	+	+	+	−	−	−	−
M-03	A	food	−	−	+	+	+	+	+	+	+	+	−	−	−	+	−	−	−	−	−	−	−	+
M-04	A	food	−	−	+	+	+	+	+	+	+	+	+	+	+	+	+	+	+	+	+	+	−	+
M-06	A	food	−	−	+	+	+	+	+	+	+	+	+	−	+	+	−	+	+	+	−	−	−	−
M-07	A	food	−	−	+	+	+	+	+	+	+	+	+	−	+	−	−	−	+	+	−	−	−	+
M-08	A	food	−	−	+	+	+	+	+	+	+	+	+	−	+	−	−	−	+	+	−	−	−	+
TM111-C1	A	food	+	−	+	+	+	+	+	+	+	+	+	+	+	+	+	+	+	+	+	+	+	−
TM111-C6	A	food	−	−	+	+	+	+	+	+	+	+	+	+	+	+	+	+	+	+	+	+	+	+
TM138C1A	A	food	+	−	+	+	+	+	+	+	+	+	+	+	+	+	+	+	+	+	+	+	+	−
TM178C5	A	food	+	−	+	+	+	+	+	+	+	+	+	+	+	−	+	+	+	+	+	+	+	−

The carriage of twelve known (from genome sequencing) *C. perfringens* chromosomal genes was first evaluated in this PCR survey. For eight representative housekeeping genes, PCR amplified a product of the expected size from all investigated *C. perfringens* strains. However, PCR for the *lonB* ORF gave negative reactions in a few investigated strains, indicating either that these strains lack *lonB* or they have minor nucleotide diversity on the primer site(s). Similarly, PCR reactions for three chromosomal genes (*eno*, *virS*, and *pfoA*) failed to amplify PCR products from chromosomal *cpe* strains that originated in Europe, Japan, or the USA, as well as from some plasmid *cpe*-positive strains and *cpe*-negative strains. These results indicate that *eno, virS* or *pfoA* genes are either missing from these strains or there is nucleotide diversity at the primer binding site(s).

PCR assays were then performed to detect the carriage of representative genes present on four completely sequenced *C. perfringens* plasmids, including two *cpe*-encoding conjugative plasmids (pCPF4969 and pCPF5603) [14], a beta2 toxin gene-encoding plasmid (pCP13) [9], and a UV inducible bacteriocin gene-encoding plasmid (pIP404) [15] (Table 1). PCR amplified products of the expected size for three *cpe*- plasmid genes (*tcpF*, *tcpH*, and *rep*) from all surveyed plasmid-*cpe* positive strains, including sporadic diarrhea strains, food poisoning strains, and food strains, as well as from several isolates originating from feces of healthy humans, and several food isolates. However, PCR assays for these genes did not produce a positive reaction from any surveyed chromosomal *cpe* strain, except for food strain P-1/09/03. In contrast, a PCR survey for three genes present on pCP13 (*soj*, *parB*, and *topA*) amplified a positive reaction for ten of eleven chromosomal *cpe* strains and for eleven of twelve plasmid *cpe* strains. In PCR assays for genes present on both *cpe* plasmids and pCP13, a *cpb2* product was not amplified from any chromosomal *cpe* strains, but a *cna* product was obtained for two chromosomal *cpe* food strains. A PCR assay for the bacteriocin gene (*bcn*) present on pIP404, was positive for 16 of 58 investigated *C. perfringens* strains (including both *cpe*-positive and *cpe*-negative strains).

### Southern blot assay for carriage of chromosomal and plasmid-borne genes

To definitively establish the presence or absence of *eno*, *virS*, *pfoA tcpH*, *cna*, and *soj* ORFs Southern blot assays were performed (Table 2) using ten chromosomal *cpe* strains, twelve plasmid *cpe* strains, two *cpe*-negative strains, and also a *Clostridium tertium* food isolate (as a negative control).

**Table 2:** The results of Southern blot assay with seven genes.

strain			*cpe*	*eno*	*virS*	*pfoA*	*tcpH*	cna	*soj*
NCTC8239	A	food poisoning	c	+	+	−	−	−	+
NCTC8798	A	food poisoning	c	+	+	−	−	−	−
OSA1	A	food poisoning	c	+	+	−	−	−	+
OSAKA2	A	food poisoning	c	+	+	−	−	−	+
OSAKA4	A	food poisoning	c	+	+	−	−	−	+
W4232	A	food poisoning	c	+	+	−	−	−	+
W5837	A	food poisoning	c	+	+	−	−	−	+
W6205	A	food poisoning	c	+	+	−	−	−	+
P-1/09/03	A	food isolate	c	+	+	−	−	−	+
T-1/08/03	A	food isolate	c	+	+	−	−	−	+
F4969	A	sporadic diarrhea	p	+	+	+	+	+	−
F5603	A	sporadic diarrhea	p	+	+	+	+	+	+
No.2	A	food poisoning	p	+	+	+	+	+	+
No.24	A	food poisoning	p	+	+	+	+	+	+
No.110	A	food poisoning	p	+	+	+	+	+	+
T1	A	food poisoning	p	+	+	−	+	+	+
T16	A	food poisoning	p	+	+	−	+	+	+
T102	A	food poisoning	p	+	+	−	+	+	+
TM111-C1	A	food isolate	p	+	+	+	+	+	+
TM138	A	food isolate	p	+	+	+	+	+	+
TM178	A	food isolate	p	+	+	−	+	+	+
MR2-4	A	human feces isolate	p	+	+	−	NT	NT	NT
MR2-12	A	human feces isolate	−	+	+	+	+	+	−
M-08	A	food isolate	−	+	+	−	−	+	−
*Clostridium tertium*		food isolate	−	+	−	−	−	−	−

NT: not tested

**Figure 1:**
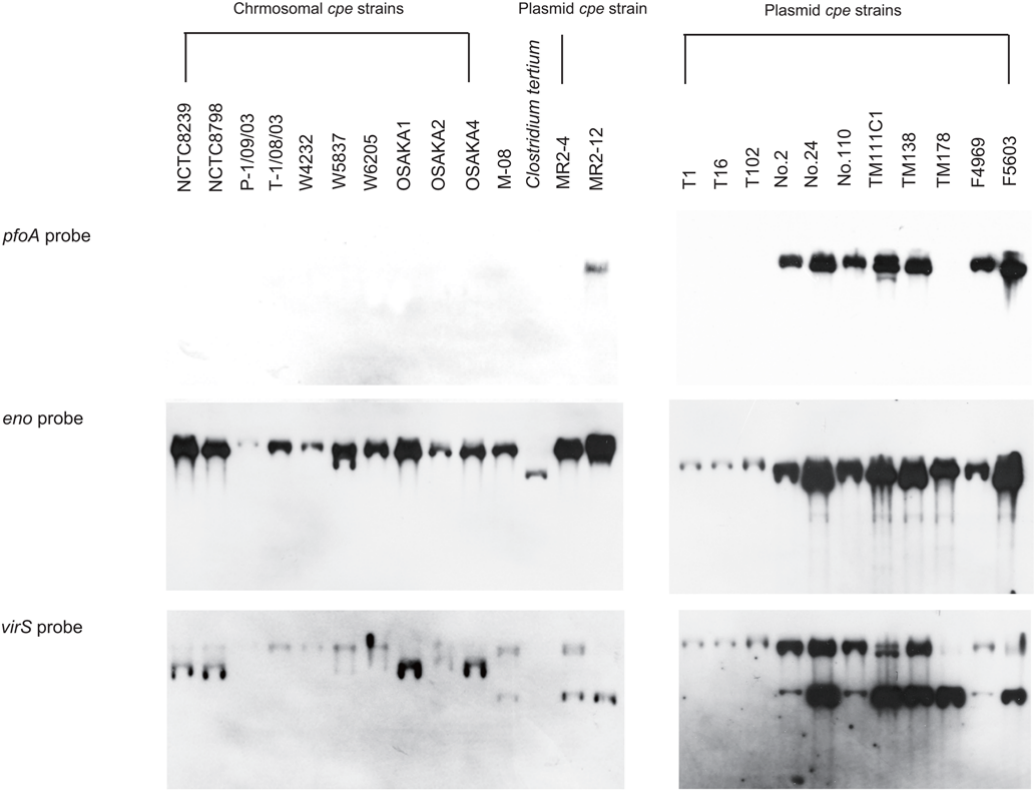
Southern blot assay of chromosomal- or plasmid-*cpe* strains. DNA digested with *Pst*I from *cpe*-positive and *cpe*-negative (M-08 and MR2-12) type A strains was subjected to 1% agarose electrophoresis prior to Southern blotting and hybridization with a DIG-labeled, *pfoA*-, *virS*-, or *eno*-specific probe.

For two tested housekeeping genes (*eno* and *virS*), Southern blot assays showed a positive reaction with all tested *C. perfringens* strains (Fig. 1). Interestingly, the positive results obtained with both PCR and Southern blot assays suggest the *eno* gene of *C. tertium* was homologous with the *C. perfringens eno* gene.

However, Southern blot assay for *pfoA* showed a negative result for all ten chromosomal *cpe* strains and for four of eleven plasmid *cpe* strains (Fig. 1), all of which also showed negative results for *pfoA* in our PCR assay.

### PCR assay for the *pfoA* region in *pfoA*-negative, *cpe*-positive strains

**Figure 2:**
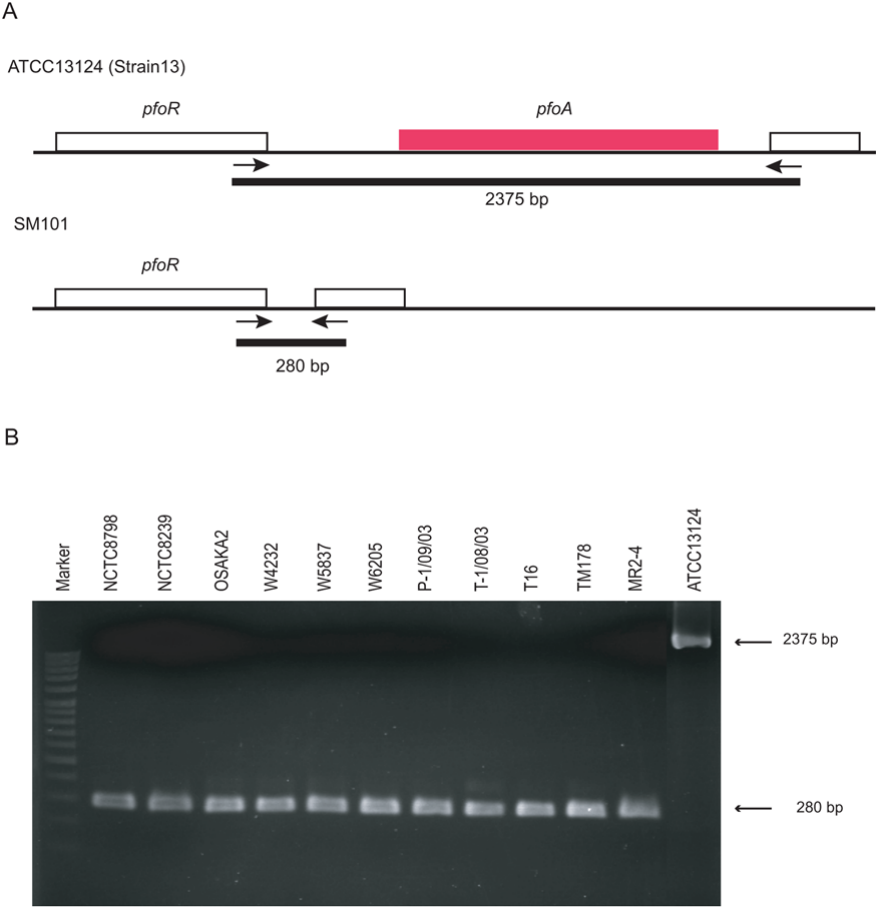
PCR assay for the *pfoA* region in *pfoA*-negative, *cpe*-positive strains. (A) Schematic representation of the *pfoA* region in *C. perfringens*. Genetic organization of the *pfoA* region is shown for *pfoA*-positive, *cpe*-negative ATCC13124 and *pfoA*-negative, *cpe*-positive SM101 strains (accession number: CP000312, and NC008261). The arrows depict the position of primers for the *pfoA* genotyping assay. The black bars show the predictive PCR products. (B) PCR results of *cpe*-positive, *pfoA*-negative strains investigated by the *pfoA* PCR genotyping assay. An ∼2,375 bp PCR product was obtained by *pfoA*-positive, *cpe*-negative ATCC13124 strain. An ∼280 bp PCR products was obtained from *pfoA*-negative, *cpe*-positive strains.

To investigate whether any portion of the *pfoA* region is still present in *cpe*-positive, *pfoA*-negative strains, we first performed a bioinformatics comparison between the recently completed sequences of *C. perfringens* strains SM101 (*pfoA*-negative) and ATCC13124 (type strain, *pfoA*-positive) [10]. This analysis indicated that the upstream *pfoR* gene and a gene downstream of *pfoA* that encodes a conserved hypothetical protein are present on the chromosome of both strains (Fig. 2A).

To further investigate the *pfoA* region amongst *pfoA*-negative, *cpe*-positive strains, primers to *pfoR* or the downstream gene were constructed for use in a *pfoA*-region genotype PCR assay. In this assay, when *pfoA* is simply deleted from the chromosome of a strain, the PCR product should be 280 bp. With this *pfoA*-genotype PCR assay, a 280 bp product was detected for eleven of twelve *pfoA*-negative, *cpe*-positive strains (Fig. 2B). These results indicate that portions of the *pfoA* region are conserved even among *pfoA*-negative type A enterotoxigenic *C. perfringens* strains, including both chromosomal *cpe* strains and plasmid *cpe* strains. Interestingly, type C human necrotizing enteritis *cpe*-positive strain, NCTC8081, also did not produce any specific *pfoA* region PCR products (data not shown).

### Multi-locus sequence typing (MLST) analysis of *C. perfringens*

The results from our PCR survey and Southern blot assay suggested that chromosomal *cpe* strains might share a common genetic background. To further compare the genetic backgrounds of chromosomal *cpe* strains versus plasmid *cpe* strains collected from various geographical origins (Japan, Europe and the US) or sources (food, food poisoning, and nonfdoodborne diarrhea patients), MLST analysis was performed with eight representative housekeeping genes.

**Figure 3:**
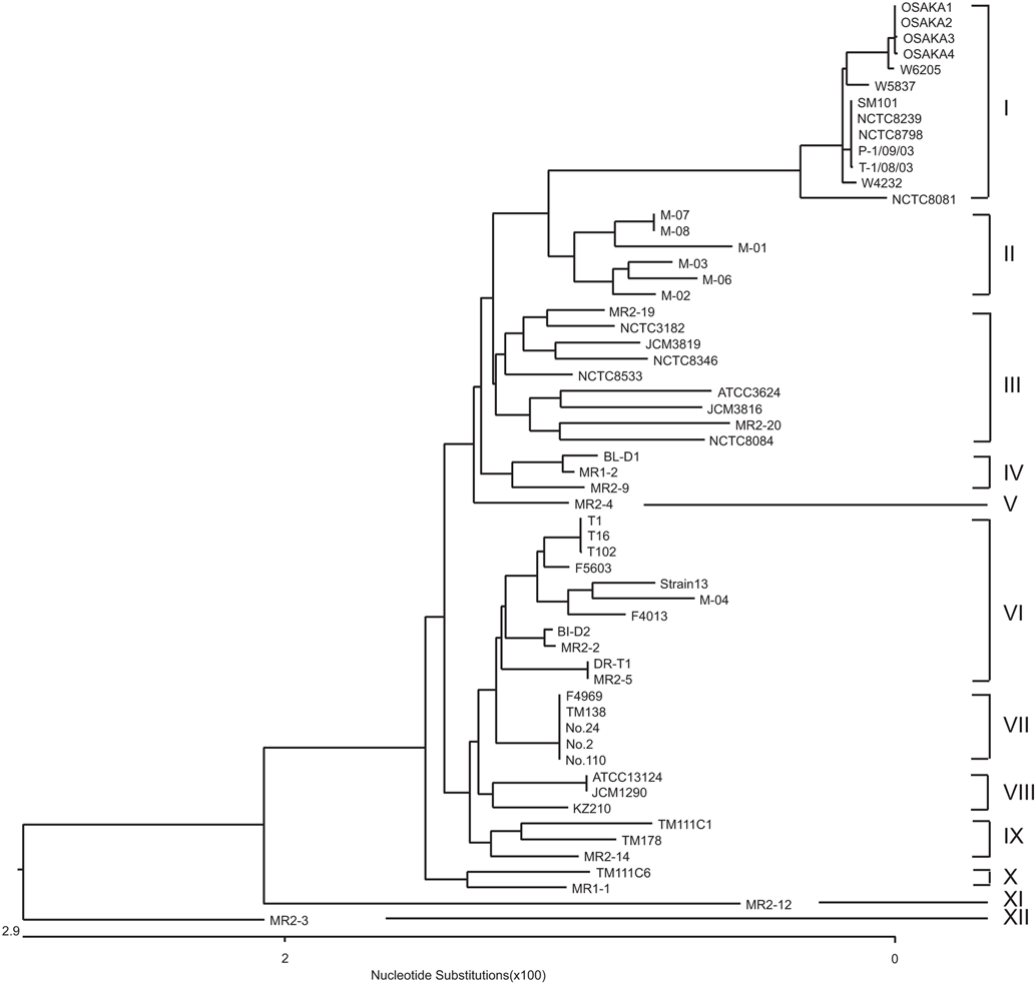
Phylogenetic relationships among 58 *cpe*-positive or *cpe*-negative *C. perfringens* strains. The phylogenetic tree was constructed by Clustal W analysis based on the concatenated nucleotide sequence of eight housekeeping genes. The phylogenetic clusters are given on the right.

Phylogenetic analysis by the Clustal W program was then performed on our MLST results for fifty-eight strains, including eleven chromosomal c*pe* strains and thirteen plasmid *cpe* strains. This analysis identified twelve main groups, designated Cluster I to XII, (Fig. 3). Several strains showed completely conserved sequences for all eight genes surveyed in this MLST. Of these strains, OSAKA1 to 4 were each isolated from the same outbreak and had been reported to show the same PFGE pattern [16]. T1, T16, and T102 strains were also isolated from a single food poisoning outbreak [17], as were No.2, No.24, and No.110 strains [18]. These MLST results suggest that strains isolated in three different food poisoning outbreaks share the same genetic backgrounds. JCM1290 is a derivative of ATCC13124 (a *cpe*-negative type strain), while SM101 is a derivative of European food poisoning isolate NCTC8798. M-07 and M-08 strains were isolated from the same meat sample so these pairs of strains might also share the same genetic backgrounds. However, thirty-seven strains gave unique individual MLST patterns in this study. Collectively, these results confirm that MLST is useful to investigate the genetic relationship between *C. perfringens* strains.

Nine chromosomal *cpe* strains were assigned to Cluster I (Fig. 3). These strains included two European food poisoning strains (NCTC8239 and NCTC8798, both isolated in the 1950s) and several Japanese food poisoning strains, including: OSAKA1, OSAKA2,OSAKA3,and OSAKA4 (each isolated in the Osaka area in 1997), W4232 (isolated from the Kanto area in 1995), and W5837 and W6205 (isolated from the Kanto area in 2000). Cluster I also included two American chromosomal *cpe* isolates (P-1/09/03,T-1/08/03) obtained from retail foods not associated with a food poisoning outbreak. These genetic findings showing relatedness between chromosomal *cpe* nonoutbreak food strains and chromosomal *cpe* food poisoning strains were consistent with previous findings indicating that food strains carrying *cpe* on the chromosome form similarly heat resistant spores as do food poisoning strains [19]. The conserved nucleotide diversity of six genes was also evident at the translational level, where there were one (*pgk*, *sod*, *gyrB*), two (*nadA*), or four (*plc*, *sigK*) amino acid substitutions. Overall, our MLST results strongly suggest that, regardless of their geographical origins, date of isolation, or origination from nonoutbreak foods or food poisoning outbreaks, type A chromosomal *cpe* strains in Japan, Europe, and USA share a common genetic background and belong to a cluster distinct from most other *C. perfringens* isolates. The one exception was that Cluster I also includes the type C *cpe*-positive NCTC8081 strain that was isolated from human necrotizing enteritis in Europe.

In contrast to the chromosomal *cpe* isolates localizing to Cluster I, three isolates from a 2001 food poisoning outbreak in Toyama area (T1,T16,and T102), each carrying a plasmid with an IS*1151* sequence downstream of the *cpe* gene [4], were assigned to Cluster VI (Fig. 3). This Cluster VI also included other plasmid *cpe* strains, with a downstream IS*1151* sequence, which had been obtained from human sporadic diarrhea cases occurring in Europe (F5603, and F4013) [20]. These findings could suggest that plasmid *cpe* isolates with downstream IS*1151* sequence share a common genetic relationship. However, this Cluster VI also included two *cpe*-negative strains.

Three food poisoning isolates from a 1980 outbreak occurring in the Toyama area of Japan (No2,No24,and No110), each carrying a plasmid with an IS*1470*-like sequence downstream of the *cpe* gene, belonged to Cluster VII, a neighboring cluster to Cluster VI (Fig. 3). This Cluster VII also included two other isolates with an IS*1470*-like *cpe* plasmid (F4969 and TM138). These two other *cpe* plasmid strains have the exact same sequence for the eight investigated housekeeping genes as found in the three Toyama food poisoning strains belonging to this cluster. This finding strongly suggested that strains in Cluster VII also share a similar genetic background.

Another three plasmid *cpe* strains (including two nonoutbreak food isolates [TM111C1, and TM178] and one plasmid *cpe* human feces isolate, MR2-4) belonged to different clusters. This genetic variability may have resulted from the conjugative nature of *cpe*- plasmids, with this plasmid transferring into unrelated *C. perfringens* strains.

Out of eleven nonoutbreak food isolates from Wakayama city in Japan that were investigated in this study (Table 1), eight isolates did not carry *cpe*. Six of those eight isolates (M-01,M-02,M-03,M-06,M-07,and M-08) belonged to Cluster II, one isolate (M-04) aligned with Cluster VI, and the final isolate (TM111C6)belonged to Cluster X. While six of these food isolates were assigned to Cluster II, no plasmid *cpe* strains from food belonged to Cluster II or VI. This finding further suggested that plasmid *cpe*-positive strains often share a similar genetic background.

Veterinary toxigenic type B to E strains (type B: NCTC8533, type C: NCTC3182,type D: NCTC8346,and type E: NCTC8084) isolated from European animals suffering from *C. perfringens* diseases were assigned to Cluster III (Fig. 3). Cluster III also included MR2-19, obtained from the feces of a healthy human, but not any meat isolates. This result might suggest that *C. perfringens* strains acting as animal pathogens have a relatively specific genetic background, but further study of additional type B-E strains is warranted.

While many strains from each source belonged to the same cluster, eleven fecal strains isolated in 2000 from healthy people in Japan belonged to eight different clusters (Fig. 3). Two isolates (MR2-19,MR2-20) belonged to Cluster III, two isolates (MR1-2,MR 2-9) aligned with Cluster IV, MR2-4 with *a cpe* plasmid carrying a downstream IS*1470*-like sequence was assigned to Cluster V, two isolates (MR2-2,MR 2-5) belonged to Cluster VI, MR2-14 aligned with Cluster IX, MR1-1 was assigned to Cluster X, MR2-12 belonged to Cluster XI, MR2-3 to Cluster XII. These findings suggested that healthy humans carry *C. perfringens* strains with various genetic backgrounds.

## Discussion

*Clostridium perfringens* strains producing enterotoxin (CPE) are the causative agent for several human GI diseases, including food poisoning, antibiotic-associated diarrhea, and sporadic diarrhea [2]. The gene encoding CPE (*cpe*) is found in a small population of type A *C. perfringen*s (approximately 1 to 5%) [2], where it can reside on the chromosome or large transferable plasmids [3], [20]. Strains carrying *cpe* on the chromosome usually possess higher resistance properties against heat, cold, and nitrates than strains carrying *cpe* on a plasmid [6]–[8]. In addition, the chromosomal *cpe* strains typically grow faster at optimal temperature, and have a broader growth temperature range, compared to plasmid *cpe* strains or other *C. perfringens* isolates [8]. These complex differences in biological properties, which are likely relevant for foodborne disease, may reflect broad genetic variations between chromosomal *cpe* isolates and other *C. perfringens* isolates.

Therefore, the current study investigated the genetic background of type A enterotoxigenic *C. perfringens*, by surveying fifty-eight *cpe*-positive and *cpe*-negative strains from various sources. Previous genetic studies have identified four groups of type A enterotoxigenic *C. perfringens*: i) food and food poisoning isolates that carry *cpe* on the chromosome [2], [19], ii) isolates that carry a plasmid-borne *cpe* gene with a downstream IS*1470-*like sequence [2], [20], iii) isolates that carry a plasmid-borne *cpe* gene with a downstream IS*1151* sequence [2], [20] and iv) isolates that carry a *cpe* gene but produced no PCR product with a *cpe*-genotyping PCR assay [21].

The results of our PCR survey and Southern blot assay for toxin genes (*plc*, *colA*, *pfoA*), remarkably found the θ toxin gene (*pfoA*) is missing from all ten surveyed chromosomal *cpe* strains and from five of twelve plasmid *cpe* strains. A study conducted back in the 1960's [22] had identified some PFO-negative, heat-resistant enterotoxigenic strains of *C. perfringens*, but localization of the *cpe* gene to the chromosome or plasmids was not possible at that time. Our finding that most chromosomal *cpe* isolates are *pfoA-*negative, significantly extends the genome sequencing observation that chromosomal *cpe* isolate SM101 is *pfoA*-negative [10].

However, our PCR analyses also indicated that the region that normally flanks *pfoA* is still present in *cpe*-positive, *pfoA*-negative strains, except for the type C NCTC8081 strain (Fig 1A, 1B).These findings suggest that most *pfoA*-negative chromosomal *cpe*, and some plasmid *cpe* isolates appear to have undergone a specific deletion of their *pfoA* gene.

Concerning housekeeping genes, our initial PCR survey for *eno*, *virS*, and *lonB* ORFs did not amplify products from many chromosomal *cpe* strains, but did for most other strains, including plasmid *cpe* strains. The presence of *eno* and *virS* genes in chromosomal *cpe* strains was confirmed by Southern blot assay (Table 2, Fig. 1). PCR surveys for genes present on the representative plasmids pCPF4969, pCP13, or pIP404, suggested that almost all chromosomal *cpe* strains carry a pCP13 and/or pIP404 related plasmid(s), but not a plasmid related to *cpe* plasmids such as pCPF4969.

In epidemiological studies of *C. perfringens* food poisoning, the relationships among *C. perfringens* isolates have primarily been investigated by pulsed-field gel electrophoresis (PFGE) [16], [23]. Although useful, PFGE lacks the precision of MLST, in which gene fragments are amplified and sequenced from several loci spread across the whole genome [11]–[13]. Three previous studies of *C. perfringens* strains using MLST analysis have been reported [24]–[26]. Two of those studies focused on *C. perfringens* animal disease isolates (type B to E) [25], [26]. The other MLST report examined a broad range of *C. perfringens*, isolates, including a limited number of *cpe*-positive food poisoning strains and suggested that food poisoning isolates form a distinct cluster of *C. perfringens* isolates.

However, that previous study examined only a few food poisoning isolates, mostly American and all carrying a chromosomal *cpe* gene, as well as only three plasmid *cpe* isolates, all nonfoodborne GI disease isolates from Europe. Since that earlier study, it has become clear that some food poisoning isolates carry their *cpe* gene on large transferable plasmids rather than on the chromosome and that chromosomal *cpe* isolates can be recovered from some retail meats. Therefore, MLST analysis using housekeeping genes present on the chromosome and *cpe* plasmid was employed in the current study to explore i) the relatedness among a larger collection of *cpe*-positive isolates, including many from Japan and ii) the similarity of these isolates to *cpe*-negative isolates. The *cpe* location, ability to produce CPE and spore heat resistance of all *cpe*-positive strains included in this study had been previously determined [6], [19], [27]. Our MLST scheme with eight housekeeping genes involves 5,274 bp (0.17% of the genome) of analyzed sequence versus the 3,918 bp of sequence included in the previous MLST applied to some *cpe*-positive *C. perfringens* [24]. Moreover, our MLST includes several genes likely contributing to survival (*sod*, and *groEL*) and propagation (*plc*, *colA*, *pgk*, *nadA*) in foods, and genes related to spore formation (*sigK*), which can also contribute to survival in foods.

From our MLST analysis, the most remarkable finding was that, regardless of their source their geographic origin, or date of isolation, all surveyed chromosomal *cpe* strains share a common genetic background and belong to the distinct Cluster I. In particular, these surveyed chromosomal *cpe* strains all possess three common features, 1) absence of the *pfoA* gene, but retention of neighboring sequences in the *pfoA* locus, 2) lack of plasmid-borne major-toxin genes (including *cpe*) [28]–[30], although they sometimes carried a plasmid encoding the putative toxin CPB2 [9], and 3) the presence in many housekeeping genes of conserved nucleotide differences, often resulting in amino acid substitutions, compared to the homolog genes present in other plasmid *cpe*-positive and/or *cpe*-negative strains.

Interestingly, cluster I also included type C strain NCTC8081, which carries *cpe* and was isolated from a patient suffering from necrotizing enteritis (Pigbel) in Europe. Human necrotizing enteritis is a rare disease and not fully understood with respect to its pathogenesis, although the plasmid-encoded β-toxin clearly plays a major role in the enteric virulence of type C isolates [31]. For research, relatively few *cpe*-positive type C strains are available from strain collections in Japan, USA, and Europe. Further investigation, if possible, using more *cpe*-positive type C strains should evaluate the genetic relationship between type A chromosomal *cpe* strains and type C *cpe*-positive strains.

Cluster VI included five strains carrying a plasmid with IS*1151* sequences located downstream of the *cpe* gene. These isolates included three food poisoning strains from an outbreak in Japan in 2000 [4], and two strains isolated from the patients with sporadic diarrhea in Europe in 1900s [20]. Of these five strains, four strains showed a very close relationship upon MLST analysis, suggesting these IS*1151 cpe*-genotype strains share a similar genetic background that facilitates their ability to cause human GI disease.

Of eight surveyed strains carrying a plasmid with IS*1470*-like sequences downstream of the *cpe* gene, five strains belonged to Cluster VII. However, the other three strains belonged to Cluster V or Cluster IX. These results suggested that while IS*1470*-like *cpe*-genotype strains often share a similar genetic background, they are more variable than IS*1151*-genotype plasmid strains.

Collectively, our results suggest for plasmid *cpe* isolates that, 1) food poisoning outbreaks involving plasmid *cpe* strains often involve clonal expansion rather than plasmid transfer, 2) IS*1151 cpe* genotype strains are closely related, but can also share a genetic relationship with some *cpe* negative isolates which may have lost the *cpe* plasmid or which represent potential future hosts for the IS*1151 cpe* plasmid, 3) IS*1470*-like *cpe* genotype strains also share genetic relationships but are more variable than the IS*1151 cpe* genotype strains. In addition, the current results conclusively demonstrate that chromosomal *cpe* isolates, whether originating from food poisoning or nonoutbreak-associated foods, do not share a close genetic linkage with plasmid *cpe* food poisoning strains (or other plasmid *cpe* strains).

Strains isolated from feces of healthy humans were found to distribute into many clusters, i.e. these strains have varied genetic backgrounds. This variability of fecal strains might be attributable, in part, to dietary differences or personal factors such as age or economic status. The investigated type B to E livestock origin strains all formed one cluster, which was distinguished from *cpe*-positive human strains (even from a type C human strain), and also from *cpe*-positive and *cpe*-negative food strains. Further MLST analyses of non-type A strains is warranted to confirm these conclusions.

Finally, the common and distinct genetic background of chromosomal *cpe* isolates provides one explanation for previous phenotypic studies [6], [7] that revealed substantial differences between the vegetative cells and spores of chromosomal *cpe* isolates versus other *C. perfringens* isolates. A shared genetic background by most or all chromosomal *cpe* isolates is also consistent with our previous studies identifying a variant small acid soluble protein, that is made by most chromosomal *cpe* isolates [32]. Regarding the evolution of chromosomal *cpe* isolates, it has been proposed [33] that these bacteria arose from integration of a composite transposon named Tn*5565* onto the *C. perfringens* chromosome. If so, our MLST findings could suggest this chromosomal transposon integration occurred only a limited number of times (perhaps only once) in a *C. perfringens* type A isolate(s) possessing a genetic background favorable for growth and survival in the food environment. Acquiring the ability to produce a potent enterotoxin, upon Tn*5565* integration, thus created a formidable food poisoning agent. Since *C. perfringens* isolates multiply to very high levels in the GI tract during food poisoning, t is possible that acquiring the ability to produce CPE is advantageous by facilitating the dissemination of these bacteria so the infectious cycle can be repeated in other hosts.

## Materials and Methods

### Bacterial strains

Fifty-eight strains of *C. perfringens* were included in this study, including type A *cpe*-positive strains, type A *cpe*-negative strains and type B to E strains. A breakdown of *C. perfringens* strains with their various origins is shown in Table 3. Briefly, the investigated strains included the type strain, several reference strains and type A chromosomal *cpe*-positive strains from food poisoning outbreaks occurring in Japan [16] or Europe [20], and isolated from foods in USA [19]. In addition, the surveyed type A plasmid *cpe*-positive strains were isolated from food poisoning outbreaks in Japan [4], [17], [18], from foods in Japan [27], from patients with sporadic diarrhea in Europe [20], or from feces of healthy humans in Japan [34]. Type B to E reference strains (one human necrotizing enteritis strain and four animal strains) were provided by NCTC.

**Table 3:** *Clostridium perfringens* strains used in this study.

	type	location of *cpe*	source	Date and region	Reference
Strain 13	A	*cpe* negative	Gas gangrene		9)
ATCC13124	A	*cpe* negative	type strain		10)
JCM1290	A	*cpe* negative	ATCC13124 derivative		this study
ATCC3624	A	*cpe* negative	Gas gangrene		
JCM3816	A	*cpe* negative	ATCC3624 derivative		this study
JCM3819	A	*cpe* negative	ATCC3629 derivative		this study
KZ210	A	*cpe* negative	BP6K derived	1940s, USA	this study
SM101	A	Chromosome	NCTC8798 derivative		10)
NCTC8239	A	Chromosome	food poisoning	1950s, Europe	21)
NCTC8798	A	Chromosome	food poisoning	1950s, Europe	21)
OSAKA1	A	Chromosome	food poisoning	1997, Japan	21)
OSAKA2	A	Chromosome	food poisoning	1998, Japan	21)
OSAKA3	A	Chromosome	food poisoning	1999, Japan	21)
OSAKA4	A	Chromosome	food poisoning	2000, Japan	21)
W4232	A	Chromosome	food poisoning	1995, Japan	21)
W5837	A	Chromosome	food poisoning	2000, Japan	21)
W6205	A	Chromosome	food poisoning	2000, Japan	21)
F5603	A	Plasmid	sporadic diarrhea	1990s, Europe	3)
F4013	A	Plasmid	sporadic diarrhea	1990s, Europe	3)
F4969	A	Plasmid	sporadic diarrhea	1990s, Europe	3)
No.2	A	Plasmid	food poisoning	1980s, Japan	18)
No.24	A	Plasmid	food poisoning	1980s, Japan	18)
No.110	A	Plasmid	food poisoning	1980s, Japan	18)
T1	A	Plasmid	food poisoning	2001, Japan	17)
T16	A	Plasmid	food poisoning	2001, Japan	17)
T102	A	Plasmid	food poisoning	2001, Japan	17)
BL-D1	A	*cpe* negative	sepsis	2001, Japan	this study
DR-T1	A	*cpe* negative	diarrhea	2001, Japan	this study
BI-D2	A	*cpe* negative	cholecystitis	2001, Japan	this study
MR1-1	A	*cpe* negative	healthy	2000, Japan	this study
MR1-2	A	*cpe* negative	healthy	2000, Japan	this study
MR2-2	A	*cpe* negative	healthy	2000, Japan	this study
MR2-3	A	*cpe* negative	healthy	2000, Japan	this study
MR2-4	A	Plasmid	healthy	2000, Japan	21)
MR2-5	A	*cpe* negative	healthy	2000, Japan	this study
MR2-9	A	*cpe* negative	healthy	2000, Japan	this study
MR2-12	A	*cpe* negative	healthy	2000, Japan	this study
MR2-14	A	*cpe* negative	healthy	2000, Japan	this study
MR2-19	A	*cpe* negative	healthy	2000, Japan	this study
MR2-20	A	*cpe* negative	healthy	2000, Japan	this study
NCTC8533	B	*cpe* negative	animal disease (lamb)	1950s, Europe	
NCTC8081	C	Plasmid	necrotizing enterocolitis	1940s, Europe	
NCTC3182	C	*cpe* negative	animal disease (sheep)	1930s, Europe	
NCTC8346	D	*cpe* negative	animal disease (sheep)	1950s, Europe	
NCTC8084	E	*cpe* negative	animal disease (calf)	1940s, Europe	
M-01	A	*cpe* negative	food isolate	2006, Japan	28)
M-02	A	*cpe* negative	food isolate	2006, Japan	28)
M-03	A	*cpe* negative	food isolate	2006, Japan	28)
M-04	A	*cpe* negative	food isolate	2006, Japan	28)
M-06	A	*cpe* negative	food isolate	2006, Japan	28)
M-07	A	*cpe* negative	food isolate	2006, Japan	28)
M-08	A	*cpe* negative	food isolate	2006, Japan	28)
TM111C1	A	Plasmid	food isolate	2006, Japan	28)
TM111C6	A	*cpe* negative	food isolate	2006, Japan	28)
TM138	A	Plasmid	food isolate	2006, Japan	28)
TM178	A	Plasmid	food isolate	2006, Japan	28)
P-1/09/03	A	Chromosome	food isolate	2003, USA	19)
T-1/08/03	A	Chromosome	food isolate	2003, USA	19)

### Bacterial culture and DNA preparation

An aliquot of a cooked meat medium [Difco] stock culture of each *C. perfringens* strain was inoculated into 5 ml of fluid thioglycolate medium(FTG [Becton Dickinson]) and then incubated overnight at 37°C. An aliquot of that overnight FTG culture was inoculated into 10 ml of TGY broth (3% Trypticase soy [Difco],2% D-glucose [WAKO],1% yeast extract [Difco],0.1% L-cystein) and then incubated overnight at 37°C. DNA for PCR and multilocus sequence typing analysis was prepared from 200 µl of overnight TGY culture with the InstaGene matrix kit [Bio-Rad] according to the manufacture's instructions. To reduce the chance of cross-contamination, DNA templates was prepared with the InstaGene matrix kit, because it requires only two tubes with three steps for preparing DNA templates. DNA materials for Southern blot assays were prepared according to methods described previously [20].

### PCR survey for housekeeping genes on the chromosome or on plasmids carrying *cpe* and/or *cpb2*

For this PCR survey, eleven housekeeping genes were selected, including: phospholipase C (alpha toxin) gene (*plc*, a ubiquitous gene of *C. perfringens*), DNA gyrase B gene (*gyrB*), one of the sporulation sigma factors (*sigK*, involved in regulating *C. perfringens* enterotoxin production [35]), three stress response genes (superoxide dismutase gene (*sodA*), heat shock protein gene (*groEL*, *lonB*), genes encoding enzymes involved in energy production from glucose (phosphofructokinase gene; *pgk*, enolase gene; *eno*), a nucleotide metabolism gene (*nadA*, quinolinate synthetase), a collagenase gene (*colA*, a possible virulence gene), a regulator gene (*virS*, a two component regulator gene) and also theta toxin gene (*pfoA*).

For plasmid-encoded genes, this PCR survey tested for: two toxin genes (the CPE gene (*cpe*) and the beta2 toxin gene (*cpb2*)), a putative collagen adhesion protein gene (*cna*) [9], plasmid transfer genes for *cpe*-carrying and antibiotics-resistant gene-carrying plasmid (*tcpF*, *tcpH*) [36], a replication gene (*rep*) on transferable plasmids [36], putative plasmid maintenance genes (*soj*, *parB*, *top*) present on pCP13, which is found in the first completely sequenced strain, strain 13 [9], and UV-induced bacteriocin gene (*bcn*) on pIP404 [15], from which most of *C. perfringens* shuttle vectors were derived.

PCR primer pairs were principally designed based on genes annotated in the *C. perfringens* strain 13 genome sequence [9], or genes on *cpe*-borne plasmids [14], or pIP404 [15] (Table 4). PCR reactions for all genes were performed under the same reaction conditions. Each PCR mixture contained 4 µl of template DNA preparations, 0.5 µl of *Taq* DNA polymerase [Promega], 2 µl of 2 µM NTP, 4 µl of 25 mM MgCl2, 5 µl of PCR buffer, 2 µl of each primer pair (1 µM final concentration). The reaction mixtures, with a total volume of 50 µl, were placed in a thermal cycler [MiniCycler, MJ] and subjected to the following amplification conditions: 1 cycle at 94°C for 2 min; 35 cycles at 94°C for 30 s, 55°C for 60 s, 68°C for 60 s, and a single extension of 68°C for 8 min. PCR products were then electrophoresed on a 1.5% agarose gel, which were stained with ethidium bromide.

**Table 4:** Primers used in this study.

Gene	Primers	Sequence (5′-3′)	Amplicon size (bp)	Analysed size (bp)	Reference
House keeping genes					
*gyrB*	gyrB-F	ATTGTTGATAACAGTATTGATGAAGC	905	735	This study
	gyrB-R	ATTTCCTAATTTAGTTTTAGTTTGCC			This study
*sigK*	sigK-F	CAATACTTATTAGAATTAGTTGGTAG	643	589	This study
	sigK-R	CTAGATACATATGATCTTGATATACC			This study
*sodA*	sod-F	CAAAAAAAGTCCATTAATGTATCCAG	663	554	This study
	sod-R	TTATCTATTGTTATAATATTCTTCAC			This study
*groEL*	groEL-F	TACAAGATTTATTACCATTACTTGAG	901	685	This study
	groEL-R	CATTTCTTTTTCTGGAATATCTGC			This study
*pgk*	pgk-F	GACTTTAACGTTCCATTAAAAGATGG	830	681	This study
	pgk-R	CTAATCCCATGAATCCTTCAGCGATG			This study
*nadA*	nadA-F	ATTAGCACATTATTATCAAATTCCTG	821	689	This study
	nadA-R	TTATATGCCTTTAATCTTAAATCCTC			This study
*colA*	colA-F	ATTAGAAAGTTTATGTACAATAGGTG	816	670	This study
	colA-R2	AAGACATTCTATTATTTCTATCGTAAGC			This study
*plc*	plc-F	AGGAACTCATGCTATGATTGTAACTC	725	671	This study
	plc-R	GGATCATTACCCTCTGATACATCGTG			This study
*lonB*	lonB-F	ATATATATGAGCAAGTCCTTTGCGAG			This study
	lonB-R	TTTTCTAATCTCTTCAACAGTTAGCC			This study
*eno*	eno-F	GCAGTACCTTCAGGAGCTTCAACAGG			This study
	eno-R	CTTCAGCCATACCATCTTCAATTGAG			This study
*virS*	virS-F	CATTGTAATAATAATTTTTTCTGTC			This study
	virS-R	TTTCCTTCAATACAGGCTATGTG			This study
*pfoA*	pfoA-F	CAAGTATTGCAATGGCTTTATGTCTG			This study
	pfoA-R	CTTTATAAGAGCTTTGAAAGCAGCTTG			This study
Genes on the representative plasmids					
*cpe*					
*can*	CAN-F	GTAGGGGAATTGATAGAACAAGACTTC			14)
	CAN-R	CTTTTATTTGAGTATCAACCATTTCAGC			14)
*cpb2*	cpb2MPRC	CAATAACCCTCACCAAATACTC			14)
	cpb2MPFC	AGATTTTAAATATGATCCTAACC			14)
*tcpF*	ORF15-HF	GACTATAGGAACTAGTGCTATAGTTGC			14)
	ORF15-HR	CGCTGGATTTACTACATAGTCCTCTG			14)
*tcpH*	ORF16-HF	GTTAATCCAGGATATGAATATTGGTGC			14)
	ORF16-HR	GTCTCTATTATAATTAGAGTTAGCAGG			14)
*rep*	repCPEF	CTTAAATCAAATCGAATATAAAGAGTC			This study
	repCPER	AATTTCTTTCTGTAAAGTTTGGTAGAG			This study
*soj*	soj-F	GGAGTTGCTAAAACAACGTCTACTGC			This study
	soj-R	CTTCAAATGTACTTTCTACTACC			This study
*parB*	parB-F	GAAATAGTGGATATTGAATCTCTTGCAG			This study
	parB-R	CCTTGTTCTATAACTGCTTTTAACTCTGG			This study
*topA*	topA-F	CATATATATTCTTGCCACAACGAGG			This study
	topA-R	GATAGTAAGATAGAAAGTCATAGTGCC			This study
*bcn*	bcnF2	GTTTCCGCCAAATGCAGTAGTAAGAG			This study
	bcnR2	GTTCATCACCAACTACCTCTGCATTG			This study
*pfoA* genotyping assay					
	pfoAR-F	AAAATACATACAGTAGATGAGATACGTGG			This study
	pfoAR-R	AAATCTGCTCTTAAAATCAATGCCTCAGC			This study

### Southern blot assays for the presence of *eno*, *pfoA*, *tcpH*, *cna*, and s*oj* genes in *C. perfringens* strains

To further investigate PCR-negative results, ten chromosomal *cpe* strains, six plasmid *cpe* food poisoning strains, three plasmid *cpe* food strains, two human sporadic diarrhea strains, were investigated by Southern blot analyses (Table 2). Plasmid *cpe* human feces isolate (MR2-4) [15], *cpe*-negative human feces isolate (MR2-12) and food isolate in Japan (M-08) were also examined. As a negative control, *Clostridium tertium* isolate, identified based on 16 rRNA gene sequence, from Japanese retail food was used.

DNA was prepared from strains F4969 or F5603 and that DNA was used with a PCR DIG-labeling kit [Roche] [34] to prepare DIG-labeled probes for *eno*, *virS*, *pfoA*, *cna*, *tcpH*, and *soj.* For Southern blot assays, DNA sample of each strains, prepared with the methods previously described [34], were digested with *Pst*I overnight at 37°C and then electrophoresed on a 1% agarose gel with 8 mA constant current, for 16 to 18 hours and then transferred to nylon membranes [Roche] with a vacuum blotter [Bio-Rad] with manufactures' instructions. The membranes were hybridized with one of the gene probes as described previously [34]. Fluorescence signals were detected with X-ray film [Fuji-Film].

### PCR analysis of the *pfoA* region in *pfoA*-negative, *cpe*-positive *C. perfringens* strains

Since PCR survey and Southern blot assay results indicated that all ten tested chromosomal *cpe* strains and five plasmid *cpe* strains do not carry *pfoA*, the genetic organization of upstream and downstream region of *pfoA* in *pfoA*-negative strains was investigated. First, a bioinformatic investigate was performed on the complete genome sequenced *pfoA*-positive and *pfoA*-negative strains, ATCC13124 and SM101 [10], respectively. From the GenBank database, both chromosomal *cpe*-positive SM101 and *cpe*-negative ATCC13124 carry upstream *pfoR* and downstream hypothetical protein gene on the *pfoA* region. To investigate whether this simple *pfoA*-specific deletion might be common among *pfoA*-negative, *cpe*-positive strains, primer pairs were constructed inside of the upstream *pfoR* ORF and a downstream hypothetical protein ORF, based on sequence information for ATCC13124 and SM101. In this *pfoA*-genotype assay, the estimated size of a PCR product should be 2,375 bp for a *pfoA*-positive strain (such as ATCC13124) but only 280 bp for a *pfoA*-negative strain (such as SM101). In this *pfoA*-genotype assay, PCR was performed with twelve *pfoA*-negative, *cpe*-positive, type A strains under the same conditions as *cpe*-genotying assay [20], but with a different *Taq* polymerase, PrimeSTAR GXL DNA polymerase [Takara], that is suitable for long-range PCR.

### Multilocus sequencing typing (MLST) analysis

To thoroughly investigate the genetic background of enterotoxigenic *C. perfringens* strains, MLST was performed using eight housekeeping genes, related to survival in foods, bacterial proliferation in foods, or spore formation (CPE is formed during sporulation). The genes used in our MLST analysis contained genes for toxin genes (*plc*, *colA*), stress response (*sodA*, *groEL*), sigma factor for sporulation (*sigK*), putative metabolism genes (*pgk*, *nadA*) and genes in DNA replication (*gyrB*). PCR products were purified with a QIA quick PCR purification kit [QIAGEN], and then sequenced with ABI PRISM®BigDye™terminator Cycle Sequencing Ready Reaction Kits (Version 1.1 and 3.1) according to the manufacture's instructions. All sequence data were concatenated to produce an in-frame 5,274 bp, according to genome arrangement of strain 13, *plc*, *colA*, *nadA*, *sodA*, *pgk*, *sigK*, *groEL*, and *gyrB*, (approximately 0.17% of strain 13 whole genome). Sequence information of these eight genes from the three completely sequenced *C. perfringens* strains, i.e., Strain13,ATCC13124,SM101 (derivative from NCTC8798), were also included in this survey. Concatenated sequence data were applied to phylogenetic analysis with Clustal W format by using Lasergene software Ver. 6 [DNASTAR].

### Nucleotide sequence accession numbers

The sequences determined in this study have been deposited in the GenBank under accession AB477535-AB477966.

Whole genome sequence information for strain 13, SM101, and ATCC13124 is available according to the following accession numbers, NC003366, CP000312, NC008261, respectively.
